# Changes of psychological and biological stress parameters in individuals with schizophrenia spectrum disorders participating in a mindfulness-based group therapy

**DOI:** 10.1038/s41537-026-00759-6

**Published:** 2026-04-30

**Authors:** Marco Zierhut, Sarah Koop, Niklas Bergmann, Inge Hahne, Ingmar Conell, Julia Kraft, Alice Braun, Mareike Bayer, Thi Minh Tam Ta, Julian Hellmann-Regen, Neil Thomas, Stephan Ripke, Malek Bajbouj, Eric Hahn, Kerem Böge

**Affiliations:** 1 Department of Psychiatry and Neuroscience, Charité – Universitätsmedizin Berlin, Berlin, Germany; 2 Berlin Institute of Health at Charité—Universitätsmedizin Berlin, BIH Biomedical Innovation Academy, BIH Charité Clinician Scientist Program, Berlin, Germany; 3 German Centre of Mental Health (DZPG) partner site Berlin, Berlin, Germany; 4 Department of Psychology, Brandenburg Medical School, Neuruppin, Germany; 5 Clinical Psychology of Social Interaction, Department of Psychology, Faculty of Life Sciences, Humboldt-Universität zu Berlin, Berlin, Germany; 6 Centre for Mental Health, Swinburne University of Technology, Melbourne, VIC Australia; 7 Stanley Centre for Psychiatric Research at Broad Institute of MIT and Harvard, Cambridge, MA USA

**Keywords:** Schizophrenia, Psychology, Psychosis

## Abstract

This study investigates changes in stress parameters in individuals with schizophrenia spectrum disorders participating in mindfulness-based group therapy (MBGT). *N* = 45 participants were randomised to MBGT or Treatment as Usual over four weeks. Before and after each session, psychological and biological stress markers by self-rating scales and cortisol saliva samples were assessed in the active group (*n* = 22). Oxytocin was assessed before and after the first and last sessions. Results indicated significant reductions in general stress, symptom-related distress and cortisol levels. Oxytocin showed increases during the first session and decreases during the last session. Exploratory analyses showed correlations between psychological and biological stress markers, and between stress reduction and changes in self-reported negative symptoms. MBGT may provide stress relief in patients with SSD with potential associations with negative symptoms. The study did not include a session-specific control group. Further studies with larger samples and corresponding control conditions are warranted to test causality.

## Theoretical background

Schizophrenia spectrum disorders (SSD) affect the lives of approximately 24 million people worldwide^1^. They count among the most severe psychiatric disorders^2^ with a lifetime prevalence of approximately 1%^3^. Treatment regimens usually include long-term medical and psychosocial support, antipsychotic medication, and psychotherapy^3^. Yet, a substantial number of patients continue to experience chronic, particularly negative symptoms^4,5^. People with SSD have higher stress levels due to several factors, and there is also evidence that more pronounced negative symptoms are associated with less effective coping, potentially contributing to increased stress^6^. Negative symptoms are one of the six core symptoms of SSD in the ICD-11 and encompass blunted affect, alogia, avolition, asociality, and anhedonia^7,8^. They are difficult to treat and remain a major contributor to poor long-term functional outcomes^9,10^.

Over the last 25 years, there has been an ever-growing research interest in mind-body interventions, with a trend for investigations starting in the early 2010s on mindfulness for individuals with SSD^11^. Mindfulness-based interventions (MBIs) were originally adapted into clinical settings as stress reduction programs that promote non-judgmental awareness of present-moment experience^11,12^. They incorporate the practice of observing and accepting thoughts, emotions, and bodily sensations as they occur, without assigning them any label or value^13^. Several meta-analyses have confirmed the overall effectiveness of MBIs in the treatment of SSD^14^, with some highlighting particularly positive effects on negative symptoms^15,16^. Within this framework, mindfulness-based group therapy (MBGT) has emerged as a relevant format for individuals with SSD. Studies have demonstrated the feasibility and clinical utility of MBGT in psychosis, with evidence pointing to reductions in negative symptom severity^17–20^. As a group-based intervention, MBGT offers broader accessibility than individual therapy, and has the potential to foster therapeutic engagement through shared experiences and social interactions—both of which are critical for addressing the social deficits inherent to negative symptoms^21^.

Stress-vulnerability models propose that the onset of SSD arises from the interaction between genetic predisposition and environmental stressors^22–24^. Individuals with a heightened vulnerability are more likely to develop symptoms when exposed to adverse experiences such as urbanicity^25^, trauma^26^, migration, or racial discrimination^27^, but also in response to everyday stressors^28^. The extended stress-diathesis model builds upon this framework by integrating both inherited and developmental factors, emphasising neuroendocrine dysregulation as a key mechanism^29–31^. Abnormalities in cortisol reactivity and hypothalamic-pituitary-adrenal (HPA) axis functioning are increasingly recognised as biological correlates of pathogenesis and stress-related symptom exacerbation in SSD^29,32,33^. To date, no studies have investigated the immediate effect of MBIs on cortisol levels in patients with SSD. Beyond cortisol, the neuropeptide oxytocin has gained attention as a potential modulator of social stress^34^. Oxytocin may buffer social stress responses^35^, and enhance social functioning^36^, both of which are relevant to the clinical profile of SSD. Previous research indicates that mindfulness practice increases oxytocin levels in healthy individuals and also in individuals with SSD^37,38^. In a previous randomised, placebo-controlled pilot study, participants with SSD received intranasal oxytocin or a placebo prior to participation in MBGT^39^. The oxytocin group showed greater improvements in negative symptom domains, along with stronger reductions in psychological stress and negative affect, compared to the placebo group^39^. Building on these results, we aimed at further investigating the impact of MBGT on endogenous oxytocin levels in SSD and its association with psychological stress parameters, cortisol and negative symptoms.

Despite the central role of stress in SSD and the growing relevance of MBGT, research on its acute effects on biological stress markers remains scarce^40,41^. Notably, no study to date has examined cortisol markers in response to MBGT in individuals with SSD. Although evidence from non-clinical populations suggests that MBIs may reduce cortisol levels^42,43^, these findings have yet to be extended to SSD. Aside from the pilot study by Zierhut et al. (2024)^39^, only one prior investigation has examined within-session changes in subjective psychological stress during MBGT in SSD^44^. To our knowledge, no study has yet combined psychological stress, cortisol, and oxytocin as concurrent outcome measures, marking a substantial gap in the literature regarding the acute stress-modulating effects of MBGT in this population.

Drawing upon previous research findings, we hypothesised that (1) subjective psychological stress levels (general stress and symptom-related distress), and (2) cortisol levels, as an objective biological stress parameter, would significantly decrease, while (3) oxytocin levels would significantly increase within MBGT sessions.

As most studies focus on changes of either psychological or biological stress in the context of MBIs, this study will analyse the correlations between various stress markers (subjective general stress, symptom-related distress, saliva cortisol, plasma and saliva oxytocin) to determine their potential interrelatedness on an exploratory level. Changes in biological stress parameters from baseline to post-intervention will be compared between the MBGT and the control group. Furthermore, the effects of MBGT on the change in negative symptoms from baseline to post-intervention will be analysed and compared between the MBGT and the control group.

## Results

### Sample characteristics

The final sample consisted of *N* = 45 participants, with *n* = 23 in the control group with treatment as usual (TAU) and *n* = 22 in the active group (MBGT + TAU). Participants were aged between 20 and 67 years, with a mean age of 43.33 years (*SD* = 12.53, *Mdn* = 42). Detailed demographic characteristics for both groups are shown in Table 1. The two groups did not differ significantly on any of the demographic variables at baseline (Table 1).

**Table 1:** Demographic characteristics of both groups at baseline.

Variable	MBGT + TAU *n* = 22	TAU *n* = 23	Statistics	*p*
	*n* (%) / mean (SD)		*χ*^2^/*t*/*W* (*df*)	
Gender			χ^2^ = 0 (1)	1
Male	13 (59%)	14 (61%)		
Female	9 (41%)	9 (39%)		
Age	44.05 (12.89)	42.65 (12.45)	*t* = 0.37 (43)	0.71
Nationality			*χ*^2^ = 2.38 (2)	0.3
German	18 (82%)	22 (96%)		
Turkish	1 (4.5%)	0		
Other	3 (14%)	1 (4.3%)		
Marital status			*χ*^2^ = 1,24 (2)	0.54
Single	16 (73%)	18 (78%)		
Married	3 (14%)	1 (4.3%)		
Separated/divorced	3 (14%)	4 (17%)		
Current housing situation			*χ*^2^ = 5.38 (3)	0.15
Private flat	18 (82%)	22 (96%)		
Shared flat	1 (4.5%)	0		
Assisted living	3 (14%)	0		
Other	0	1 (4.3%)		
Years in school^1^	12.09 (1.34)	11.75 (1.33)	*W* = 277	0.39
Highest Educational Achievement			*χ*^2^ = 6.63 (7)	0.47
Without qualification	2 (9.1%)	0		
Primary school	1 (4.5%)	0		
Lower secondary	4 (18%)	5 (22%)		
A level	2 (9.1%)	5 (22%)		
Higher secondary	1 (4.5%)	0		
Vocational training	2 (9.1%)	3 (13%)		
University degree	10 (45%)	9 (39%)		
Other	0	1 (4.3%)		
Employment status			*χ*^2^ = 1.32 (5)	0.93
Unemployed	4 (18%)	3 (13%)		
In retirement	6 (27%)	9 (39%)		
Student	2 (9.1%)	3 (13%)		
Self-employed	3 (14%)	2 (8.7%)		
Employed	4 (18%)	4 (17%)		
Other	3 (14%)	2 (8.7%)		
Diagnosis^1^			*χ*^2^ = 2.11 (2)	0.35
F20	14 (64%)	15 (68%)		
F23	2 (9.1%)	0		
F25	6 (27%)	7 (32%)		

Diagnosis refers to diagnostic categories of the International Classification of Diseases (ICD-10). F20 = Schizophrenia, F23 = acute and transient psychotic disorders, F25 = schizoaffective disorders. *P-*values are based on *Chi*-square tests for categorical and *t*-tests for continuous variables. In case of a violated normal distribution of residuals, the nonparametric Wilcoxon rank sum test was used (*W*).

*MBGT* mindfulness-based group therapy, *TAU* treatment as usual, *SD* standard deviation.

^1^Missing data (*n* = 1).

Supplementary Table 1 in the supplementary material presents the mean, standard deviation, minimum, and maximum values of negative symptoms with the positive and negative syndrome scale (PANSS) and the self-evaluation of negative symptoms (SNS) in both groups at baseline (T0) and post-intervention (T7).

Six participants in the active group and four participants in the control group obtained minor adaptations in their substance or dose of their first or second antipsychotic medication. Supplementary Table 2 in the supplementary material provides a descriptive overview of medication for each participant at baseline and post-intervention.

### Overview of changes in stress parameters within sessions over the intervention course

**Fig. 1:**
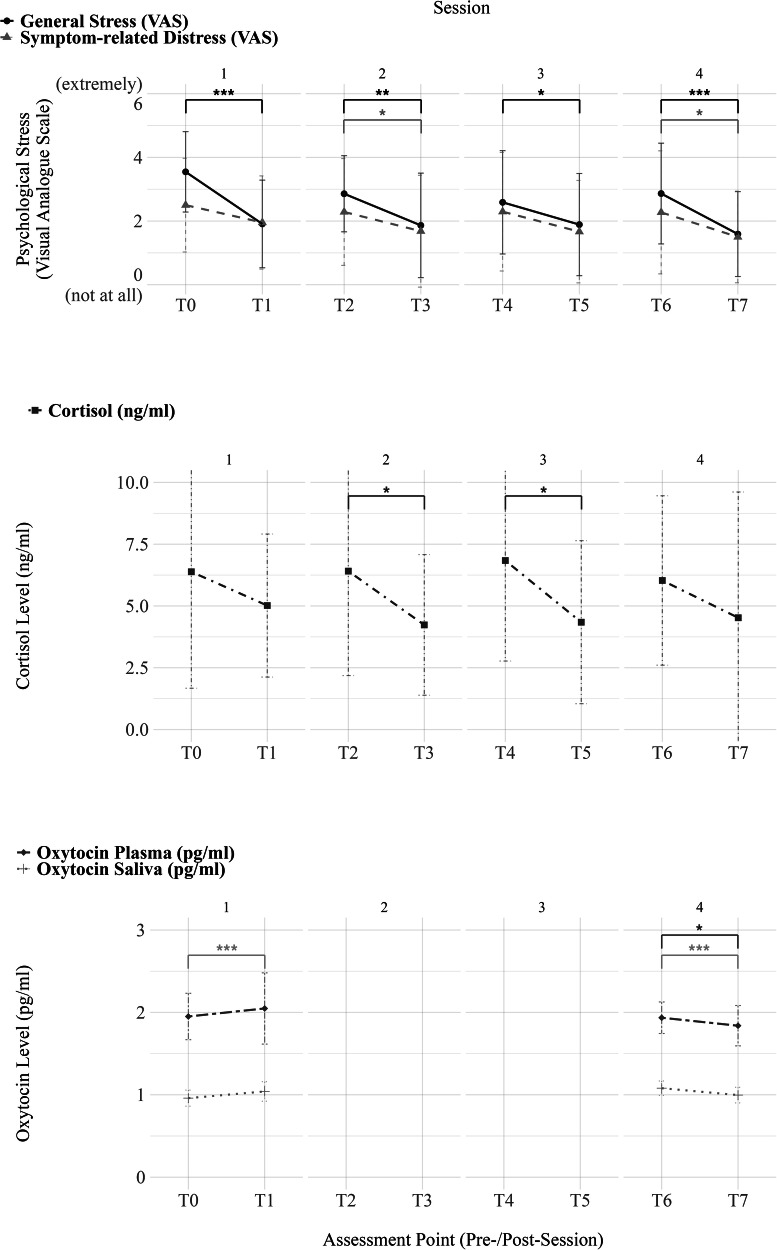
Mean within-session changes in general stress, symptom-related distress, cortisol and oxytocin in plasma and saliva in the active group (*n* = 22). Error bars depict standard deviations at each assessment point. VAS, visual analogue scale; self-report. Plasma and saliva oxytocin levels were only assessed before and after sessions 1 and 4 (T0, T1, T6, T7). Brackets and stars indicate the significance of within-session changes. ^*^p < 0.05, ^**^p < 0.01, ^***^p < 0.001.

Figure 1 and Supplementary Table 3 in the supplementary material present within-session changes in general stress, symptom-related distress and cortisol, as well as plasma and saliva oxytocin levels for the active group. Supplementary Table 4 in the supplementary material shows a summary of the model parameters of the random-intercept fixed-slope LMM for the dependent variables general stress, symptom-related distress, and cortisol.

#### Psychological stress parameters

The analysis revealed a significant pre-to-post session (fixed) effect on general stress levels across all four sessions (*b* = −1.201, b^*^ = −0.377, *SE* = 0.159, *t*(142.73) = −7.532, *p* < 0.001). On symptom-related distress, the overall pre-to-post session (fixed) effect across sessions was smaller, but still significant (*b* = −0.675, b^*^ = −0.193, *SE* = 0.154, *t*(82.52) = −4.377, *p* < 0.001). *T*-tests were calculated to assess the difference for each session individually, indicating a significant pre-to-post session decrease in each of the four sessions for general stress and in sessions 2 and 4 for symptom-related distress (Table 2). Supplementary Table 3 in the supplementary material provides an overview of mean values at each assessment point.

**Table 2:** Results of *T*-tests for session-specific pre-to-post changes in general stress, symptom-related distress, and cortisol in the active group (*n* = 22).

Variable	Session	*t*(*df*)	*p*	95%-CI [lower; upper]
General stress (H1)	1	5.937(21)	<0.001^***^	[1.063; 2.21]
	2	2.871(20)	0.009^**^	[0.312; 1.973]
	3	2.135(16)	0.049^*^	[0.006; 1.641]
	4	4.965(21)	<0.001^***^	[0.74; 1.806]
Symptom-related distress (H1)	1	1.867(21)	0.076	[−0.062; 1.153]
	2	2.169(20)	0.042^*^	[0.029; 1.495]
	3	1.890(16)	0.077	[−0.079; 1.373]
	4	2.773(21)	0.011^*^	[0.193; 1.352]
Cortisol (H2)	1	1.961(14)	0.07	[−0.162; 3.627]
	2	4.17(19)	0.001^**^	[1.256; 3.788]
	3	2.826(20)	0.01^*^	[0.654; 4.337]
	4	1.364(17)	0.19	[−0.785; 3.657]
Oxytocin—plasma (H3)	1	−1.155(20)	0.262	[−0.284; 0.081]
	4	2.566(21)	0.018^*^	[0.018; 0.175]
Oxytocin—saliva (H3)	1	−4.793(20)	<0.001^***^	[−0.111; −0.044]
	4	4.377(21)	<0.001^***^	[0.044; 0.0124]

*CI* confidence interval, *H1* hypothesis 1, *H2* hypothesis 2, *H3* hypothesis 3.

^*^p < 0.05, ^**^p < 0.01, ^***^p < 0.001.

#### Biological stress parameters (cortisol, oxytocin)

**Fig. 2:**
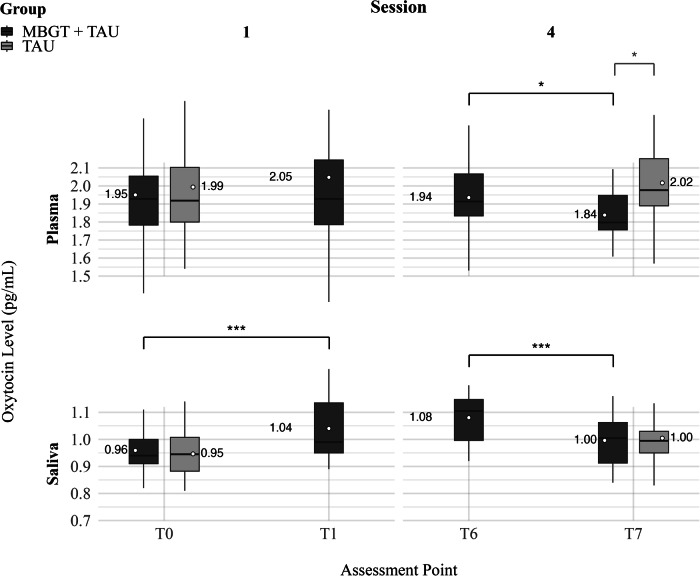
Plasma and saliva oxytocin levels at session 1 (T0 and T1) and session 4 (T6 and T7)—within session and group comparison between active group (MBGT + TAU) and control group (TAU). In the active group (MBGT + TAU), oxytocin was assessed before and after Sessions 1 and 4 (T0–T1, T6–T7). In the control group (TAU), oxytocin was assessed at baseline/before Session 1 (T0) and post-intervention/after Session 4 (T7). The *y*-axis has been truncated to enhance readability. White dots represent mean values. Box limits represent lower and upper quartiles, and black horizontal lines within the boxes represent medians. Box extension lines (“whiskers”) represent the 1.5× interquartile range. Brackets and stars indicate the significance of within-session changes within and between groups. MBGT mindfulness-based group therapy, TAU treatment as usual. ^*^p < 0.05, ^**^p < 0.01, ^***^p < 0.001.

Examining the effects on cortisol levels, there was also a significant pre-to-post session (fixed) effect across sessions (*b* = −1.99, b^*^ = −0.245, SE = 0.418, *t*(78.31) = −4.763, *p* < 0.001). *T*-tests for each session revealed significant cortisol reductions in sessions 2 and 3 (Table 2 and Fig. 1). Analysis of the oxytocin levels indicated that in plasma and saliva, levels increased within the first MBGT session and decreased within the last session (Table 2 and Figs. 1, 2).

Oxytocin saliva and plasma levels at T0 and T7 in both groups are shown in Table 3. *T*-tests revealed that while oxytocin saliva levels at T0 and T7, and oxytocin plasma levels at T0 did not differ between the groups, oxytocin plasma levels at T7 were significantly higher in TAU compared to MBGT + TAU (Table 3 and Fig. 2). Cortisol levels at T0 and T7 did not differ between the groups (Table 3).

**Table 3:** Between-group comparison of oxytocin and cortisol levels at baseline (T0) and post-intervention (T7) in both groups.

		MBGT + TAU (*n* = 22)	TAU (*n* = 23)				
		Mean (SD)	Mean (SD)	*t*	*df*	*p*	CI [lower; upper]
Oxytocin plasma (pg/mL)	T0	1.95 (0.28)	1.99 (0.33)	−0.467	40.57	0.643	[−0.284; 0.081]
	T7	1.84 (0.25)	2.02 (0.30)	−2.110	38.40	0.041^*^	[−0.350; −0.007]
Oxytocin Saliva (pg/mL)	T0	0.959 (0.098)	0.946 (0.089)	0.456	40.17	0.651	[−0.111; −0.044]
	T7	0.996 (0.095)	1.005 (0.102)	−0.277	40.46	0.783	[−0.069; 0.052]
Cortisol (ng/mL)	T0	6.38 (4.71)	4.95 (3.37)	1.00	24.19	0.327	[−1.521; 4.383]
	T7	4.52 (5.09)	5.67 (4.31)	−0.729	33.91	0.471	[−4.326; 2.041]

*MBGT* mindfulness-based group therapy, *TAU* treatment as usual, *SD* standard deviation, *CI* confidence interval.

T0 = baseline. T7 = post-intervention.

^*^p < 0.05, ^**^p < 0.01, ^***^p < 0.001.

### Correlations between stress parameters

Table 4 shows the correlation coefficients between the various stress parameters (general stress, symptom-related distress, cortisol, oxytocin plasma and oxytocin saliva). Significant correlations were observed between the two psychological stress scales, general stress and symptom-related distress (*ϱ* = 0.689*, p* < 0.001), between cortisol and both general stress (*ϱ* = 0.397*, p* < 0.001) and symptom-related distress (*ϱ* = 0.369*, p* < 0.001;), between oxytocin plasma scores and symptom-related distress (*ϱ* = 0.224*, p* = 0.037), and between oxytocin plasma and cortisol levels (*ϱ* = 0.19*, p* = 0.049). The correlations between cortisol and the psychological stress variables remained significant after conservatively using a Bonferroni–Holm correction for multiple comparisons, but other correlations dropped below significance.

**Table 4:** Correlations between different stress parameters with *p*-values before and after correcting for multiple testing.

Variable	1. Cortisol	2. Oxytocin plasma	3. Oxytocin saliva	4. General stress (VAS)
1. Cortisol				
2. Oxytocin plasma	0.190			
	[0, 0.37]			
	*p* = 0.049^*^ ***p*** = **0.588**			
3. Oxytocin saliva	0.167	−0.044		
	[−0.02, 0.34]	[−0.21, 0.13]		
	*p* = 0.086 ***p*** = **0.855**	*p* = 0.621 ***p*** = **1.0**		
4. General stress (VAS)	0.397	0.092	−0.015	
	[0.25, 0.53]	[−0.12, 0.30]	[−0.23, 0.20]	
	*p* < 0.001^***^ ***p*** < 0.001^***^	*p* = 0.398 ***p*** = **1.0**	*p* = 0.887 ***p*** = **1.0**	
5. Symptom-related distress (VAS)	0.369	0.224	0.011	0.689
	[0.22, 0.50]	[0.01, 0.42]	[−0.20, 0.22]	[0.60, 0.76]
	*p* < 0.001^***^ ***p*** < 0.001^***^	*p* = 0.037^*^ ***p*** = **0.516**	*p* = 0.922 ***p*** = **1.0**	*p* < 0.001^***^ ***p*** < 0.001^***^

Values in square brackets indicate the 95% confidence interval for each correlation. *p*-values after Bonferroni–Holm adjustment are marked in bold.

*VAS* visual analogue scale.

^*^p < 0.05, ^**^p < 0.01, ^***^p < 0.001.

### Effects of changes in stress parameters on changes in negative symptoms

Change scores across sessions for psychological stress scales (general stress = ∆gStress and symptom-related distress = ∆srdStress) and for cortisol (∆Cortisol) as a biological stress marker were calculated and used as predictor in regression analyses to examine their effect on change scores (T7–T0) of negative symptoms, both self-rated (∆NS/SNS) and clinician-rated (∆NS/PANSS) (Supplementary Table 1). The results of the individual regressions are presented in Table 5.

**Table 5:** Effects of average changes in stress levels on changes in negative symptoms from T0 to T7 in the active group (*n* = 22): results of linear regression models.

	Term	*b*	b^*^	SE	*t*	*p*	*R*^2^	Adj. *R*^2^
1(a) Effect of ΔgStress on ∆NS/PANSS	(Intercept)	1.524		1.769	0.862	0.399		
	∆gStress	2.296	0.396	1.190	1.929	0.068	0.157	0.115
1b) Effect of ΔgStress on ∆NS/SNS	(Intercept)	2.765		2.072	1.334	0.197		
	∆gStress	3.327	0.471	1.394	2.387	0.027^*^	0.222	0.183
2a) Effect of ΔsrdStress on ∆NS/PANSS	(Intercept)	0.324		1.340	0.242	0.811		
	∆srdStress	2.295	0.397	1.185	1.937	0.067	0.158	0.116
2b) Effect of ΔsrdStress on ∆NS/SNS	(Intercept)	0.738		1.624	0.454	0.654		
	∆srdStress	2.874	0.408	1.437	2.001	0.059	0.167	0.125
3a) Effect of ΔCortisol on ∆NS/PANSS	(Intercept)	0.423		1.591	0.266	0.793		
	∆Cortisol	0.887	0.303	0.625	1.419	0.171	0.092	0.046
3b) Effect of ΔCortisol on ∆NS/SNS	(Intercept)	−0.664		2.029	−0.327	0.747		
	∆Cortisol	0.243	0.068	0.798	0.304	0.764	0.005	−0.045

T0 = baseline. T7 = post-intervention. b^*^ = standardised effect.

*SE* standard error, *Adj*. adjusted, *∆gStress* averaged change in general stress across sessions, *∆srdStress* averaged change in symptom-related distress across sessions, *∆Cortisol* averaged change in cortisol levels across sessions, *∆NS/PANSS* change in clinician-rated negative symptoms between T0 and T7 assessed via the positive and negative syndrome scale, *∆NS/SNS* change in self-rated negative symptoms between T0 and T7 assessed via the self-evaluation of negative symptoms. ^*^p < 0.05, ^**^p < 0.01, ^***^p < 0.001.

While for the psychological stress parameters (general stress and symptom-related distress), a trend could be observed suggesting a linear relationship between changes in psychological stress levels and changes in clinician-rated and self-rated negative symptoms (see Table 5), with a significant effect of change in general stress on change in self-rated negative symptoms (*F*(1,20) = 5.697, *p* = 0.027), the change in cortisol showed no direct effect on the change in negative symptoms, clinician-rated (*F*(1,20) = 2.015, *p* = 0.171) or self-rated (*F*(1,20) = 0.093, *p* = 0.764).

## Discussion

The present study investigated the association between MBGT and changes in stress markers, as well as the potential link between stress regulation and negative symptoms in individuals with SSD. Hypotheses 1 and 2, that subjective psychological stress levels, and cortisol, as an objective biological stress parameter of individuals with SSD participating in an MBGT, would decrease within sessions, could be confirmed. Across all four sessions, participants in the active group exhibited significant within-session reductions in psychological stress, as assessed by the general stress and symptom-related distress subscales of a visual analogue scale^44^. This reduction was accompanied by significant decreases in cortisol, a biological marker of stress, which showed a consistent pre-to-post session decrease across sessions. Besides supporting previous findings of stress-reducing potential of MBIs for people with SSD^44^, these findings support changes in cortisol as a possible biological stress parameter with consistent within-session changes also within a brief four-week protocol, which is shorter than most other MBI trials for SSD^21^. Due to a missing active control group on a within-session basis, we are not able to make any statements about causality. These results are in line with studies supporting the value of mindfulness as a feasible tool for managing daily stressors^12^, which may contribute to relapse prevention and improved functional outcomes. While the social context of group therapy may also contribute to these effects^45^, the consistency of reductions observed across sessions was notable. Future studies should build upon these findings to investigate the causal effects of MBGT or MBIs on different stress parameters within a group comparison framework.

Another contribution of this work lies in the aspect of session-specific changes of endogenous oxytocin levels, even though hypothesis 3 could only partly be confirmed. Oxytocin levels increased during the first MBGT session but decreased in both plasma and saliva during the final session, with post-intervention plasma levels being significantly lower in the active group than in the control group^38^. In line with refs. ^35^ and ^36^, this trajectory emphasises that oxytocin may act less as a direct indicator of social stress levels and more as a regulatory hormone influenced by both social and contextual factors. In light of this, one could assume that in the first session, which is associated with heightened social stress due to the new setting and new people in the group, oxytocin is released in greater quantities as a counter-regulatory response. In the final session, however, where the setting and people are familiar and due to the stress-reducing effect of MBGT in this setting, there may have been a decrease in oxytocin. This interpretation would, however, first need to be confirmed in a larger-scale study in which more than just two within-session changes in oxytocin are examined over time.

The lack of correlation between oxytocin concentrations in plasma and saliva in our exploratory analyses may reflect temporal differences in oxytocin release and a slower accumulation of salivary oxytocin. This mismatch should be taken into account and further investigated in future studies.

In our exploratory analyses, cortisol levels were significantly correlated with self-reported stress parameters across sessions, further illuminating the interplay between biological and psychological stress systems^29^. This highlights the important relationship of these measures, which should be further explored in future research. If this interrelation can be confirmed, self-report tools could serve as a less expensive, faster, and non-invasive alternative for assessing stress in clinical practice and research. However, the absence of parallel session assessments in an active control group in the present study precludes direct attribution of the changes to mindfulness alone.

Consistent with the only partial confirmation of hypothesis 3, correlations between cortisol and oxytocin levels were low (plasma) to absent (saliva), indicating that the two hormones might represent distinct components of stress regulation. Our findings support the notion that cortisol and oxytocin capture distinct dimensions of stress regulation: while cortisol reflects classical HPA-axis-related stress physiology, oxytocin may primarily index context-dependent social-affiliative processes. Given the established role of oxytocin in social cognition and affiliative behaviour, these findings may indicate an interaction between mindfulness-induced stress regulation and social-affiliative neurobiology^45^.

Exploratory analyses also suggested that reductions in general stress over the course of the intervention might be associated with improvements in self-reported negative symptoms on the SNS but not in clinician-rated PANSS-NS scores. This discrepancy highlights the possibility that stress regulation more strongly influences patients’ subjective experiences—such as feelings of emotional disconnection or social withdrawal—than externally observed behaviour^8^. These results align with the view that group-based mindfulness interventions may support improvement in negative symptoms, partly through stress reduction in socially enriched contexts^6^.

Several limitations must be acknowledged. The modest sample size limits statistical power, and the absence of psychological stress assessments in the control group reduces the ability to draw causal inferences. Without active control conditions, especially on a session basis, it remains unclear whether observed changes are attributable to mindfulness content, group interaction, non-specific therapeutic factors, or are even an artefact of things occurring prior to the session. The four-week duration, although feasible, is shorter than most MBGT protocols targeting negative symptoms, which often span eight weeks or more^21^. Finally, oxytocin and cortisol are influenced by multiple biological and environmental factors, including diurnal variation, medication effects, and sex differences, which could not be fully accounted for in this analysis^29^.

In conclusion, this study provides preliminary indications that MBGT might produce immediate and measurable reductions in both psychological and biological stress parameters in SSD, with potential downstream effects on self-reported negative symptoms. Concurrent cortisol and oxytocin modulation suggests that mindfulness may benefit SSD through synergistic effects on stress-responsive neuroendocrine systems and social-affiliative processes. Future fully powered, multi-centre RCTs should treat the present findings as hypothesis-generating and incorporate active comparators (e.g., supportive group discussion or psychoeducation controlling for group contact and expectancy) to also be able to disentangle social vs mindfulness-specific effects. Moreover, integrating ecological momentary assessment before, during, and after the intervention (e.g., 4–6 brief prompts/day assessing stress, affect, coping, mindfulness-state, and social context) would allow modelling of within-person stress changes and help to better account for diurnal variation and momentary contextual factors (e.g., stress exposure and social context). In the long run, such work could inform targeted, multimodal interventions integrating mindfulness to address both stress and negative symptoms in SSD.

## Methods

### Study design

**Fig. 3:**
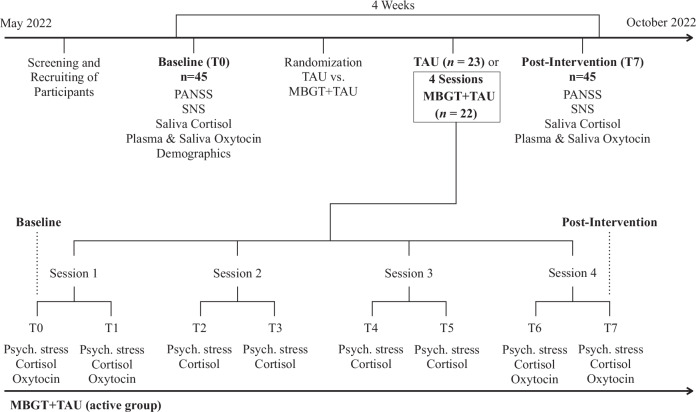
Description of the study design and assessment points of outcome measures. PANSS positive and negative syndrome scale, SNS self-evaluation of negative symptoms, MBGT mindfulness-based group therapy, TAU treatment as usual, IG intervention group, Psych. stress psychological stress, assessed with a visual analogue scale including two scales for general stress and symptom-related distress. In the MBGT + TAU group, psychological stress (general stress and symptom-related distress) and cortisol levels were assessed before and after each MBGT session (T0–T7). Additionally, plasma and saliva oxytocin levels were assessed before and after sessions 1 and 4 (T0, T1, T6, T7). In the TAU group, cortisol, as well as plasma and saliva oxytocin, was assessed at baseline and post-intervention (T0 and T7). Negative symptoms by the PANSS and the SNS were assessed at baseline and post-intervention in both groups.

Data for the present analysis originate from a rater-blinded, parallel-group, randomized and controlled study^38^. The original study was approved by the ethics committee of Charité—Universitätsmedizin Berlin (EA4/233/21) and was registered as a clinical trial in August 2022 on clinicaltrials.gov (NCT05491486). Data collection took place between May and October 2022. After baseline assessment, *N* = 45 participants were randomly assigned to either four weekly MBGT sessions in addition to outpatient treatment as usual (MBGT + TAU; active group; *n* = 22) or only outpatient treatment as usual (TAU; control group; *n* = 23). Randomisation was performed by an independent researcher using a computer-generated allocation sequence with a 1:1 ratio and fixed block size. The study design is depicted in detail in Fig. 3. Study data were collected and managed using an electronic data capturing platform, REDCap (Research Electronic Data Capture)^46,47^, hosted at Charité—Universitätsmedizin Berlin, Campus Benjamin Franklin.

### Participants

Participants were recruited from outpatient facilities, psychiatric and psychotherapeutic practices, assisted living facilities, and psychiatric day clinics in Berlin. Flyers with study information were used, as well as direct referrals from medical or psychotherapeutic practitioners. Participants were eligible to participate in the study if they (a) were aged between 18 and 67 years, (b) met diagnostic criteria for SSD (ICD-10: F2X.X) ascertained by a trained psychiatrist, (c) had sufficient German language proficiency to engage with the intervention, (d) had no recent (<4 weeks) major change in psychopharmacologic medication, and (e) were able to give written informed consent. Women further had to provide a negative pregnancy test prior to inclusion. Exclusion criteria encompassed (a) a score of 7 on any item of the positive scale of the PANSS^48^, suggesting severe psychotic symptoms, (b) acute suicidality, (c) current substance use other than nicotine, or (d) the presence of neurological disorders or brain damage. Participants were required to keep their medication stable during the 4-week intervention phase to prevent confounding of the stress assessment. Psychotropic medication and dosage were systematically recorded and checked for outliers (Supplementary Table 2). After eligibility screening, participants provided written informed consent. Participation was voluntary, and withdrawal was possible at any time during the trial without giving reasons. A CONSORT diagram of the participant flow from the original study was published in ref.^38^ and can be found in the supplementary material (Supplementary Fig. 1).

### Intervention

Participants in the active group attended weekly 60-min MBGT sessions in addition to TAU over the course of four weeks. The MBGT sessions were carried out by trained psychotherapists and based on a manual^13^ specifically developed using a participatory approach with patients with SSD, which showed high feasibility and acceptability in previous trials^17,49^. The MBGT intervention consists of four different modules, each introducing one core aspect of mindfulness: (1) breathing, (2) senses in nature, (3) detachment, and (4) body awareness^13^. After each session, participants were encouraged to formulate a mindful goal for the upcoming week and to thereby engage with mindfulness in between sessions.

### Measures

Figure 3 provides an overview of the outcome measures that were assessed at each time point. The study was originally designed as a proof-of-concept study to explore the effects of MBIs in a population of patients with SSD^38^. The original pilot design focused on pre-post intervention between-group comparisons of clinical outcome measures such as oxytocin levels and PANSS scores. Therefore, a non-active control group was considered appropriate for the initial investigation.

#### Stress

In the active group, psychological stress levels using self-rating scales and cortisol saliva samples were assessed at each time point before and after each MBGT session (T0–T7), including baseline (T0) and post-intervention (T7). Oxytocin saliva and plasma samples were collected before and after the first and last sessions in the active group (T0, T1, T6, T7). In the control group, all measurements were assessed at baseline (T0) and post-intervention (T7). An overview of the study design can be found in Fig. 3.

The reason for the lower number of oxytocin measurements was, on the one hand, that the patients did not want to have blood taken before and after each MBGT session, and also to avoid stress. In addition, the high costs of determining oxytocin levels played a role, which had to be considered in the study budget. All samples were taken at resting state at the same time at 1 pm when the participants arrived at the study site before the session and right after the 60-min session. Based on previous experience, an earlier time point for the intervention and the associated sample collection proved unrealistic in practice, as many patients refused earlier regular appointments for group therapy and would find the associated early arrival stressful, which the study setting sought to avoid.

The two self-rating scales measured general stress and symptom-related distress. They both used a novel response format (visual analogue scales), developed by ref.^44^, comprising seven circles of varying size. Each circle represents the extent of feeling stressed (in general or referring to perceived symptom burden) on a scale from 0 = *not at all* (smallest circle) to 6 = *extremely* (largest circle). Due to the single-item format and the visual aid provided by the circles, the scale was considered particularly suitable for the rapid assessment of stress within a limited time frame.

Saliva cortisol samples were collected using polypropylene blue cap Salivette® Cortisol tubes (Sarstedt, Nümbrecht, Germany) with synthetic fibre swabs, labelled with participant ID, date, and assessment point. Samples were frozen at −20 °C until analysis at the Neurobiology Laboratory of the Department of Psychiatry, Charité—Universitätsmedizin Berlin, Campus Benjamin Franklin. The analysis followed a standardised procedure routinely employed in this laboratory and previously documented^50^.

Oxytocin was measured in plasma and saliva for comparison. Saliva oxytocin samples were also collected using polypropylene blue cap Salivette® Cortisol tubes (Sarstedt, Nümbrecht, Germany) with synthetic fibre swabs. For the measurement of plasma oxytocin levels, venous blood samples (10 ml each) were obtained in Ethylenediaminetetraacetic acid (EDTA) Monovette tubes (Sarstedt, Nümbrecht, Germany) containing aprotinin 400 IU/ml blood to avoid hormone degradation. Samples were kept on ice for up to a maximum of 20 min until centrifugation at 1300×*g* for 10 min at 4 °C. Supernatants were collected and stored immediately at −20 °C for a maximum of six months until oxytocin levels (pg/ml) were analysed by a highly sensitive (0.5 pg/ml range) and specific (<0.7% cross-reactivity to a variety of peptides) radioimmunoassay with intra- and inter-assay variabilities of less than 10% (RIAgnosis, Munich, Germany)^39,51^.

#### Negative symptoms

Negative symptoms were measured before and after the full intervention (T0 and T7), rater-based and self-rated. As a rater-based instrument, the Negative Scale of the PANSS (PANSS-NS)^48^ was used. The Negative Scale comprises seven items, representing negative symptoms and assessing symptom severity on a seven-point Likert scale (from 1 = *Absent* to 7 = *Extreme*). The total score ranges from 7 to 49 points. In the present sample, internal consistency of the PANSS-NS was acceptable (*Cronbach’s α* = 0.685 at T0).

The SNS was additionally administered to assess self-rated, subjectively experienced negative symptom severity^52^. It holds five subdomains, namely (1) social withdrawal, (2) diminished emotional range, (3) avolition, (4) anhedonia, and (5) alogia. The SNS includes 20 items referring to the past week, which are rated on a three-point Likert scale (from 0 = *Strongly Disagree* to 2 = *Strongly Agree*). It showed strong internal consistency in the present sample with *Cronbach’s α* = 0.854 at T0.

### Statistical analysis

All statistical analyses were performed using R Statistical Software (v4.3.0)^53^ and R Studio (v2023.6.0.421)^54^. The sample size was established through power analyses based on the main trial outcomes reported in ref.^38^.

Immediate changes in stress levels (general stress, symptom-related distress, cortisol) within sessions were analysed using a linear mixed model (LMM) to account for the nested data structure. In the present study, pre- and post-assessment points (Level 1) were nested within sessions (Level 2), which were nested within participants (Level 3). The model was fitted using functions from the lme4 package (v1.1.33)^55^. Psychological and biological stress levels were included as dependent variables. The assessment point (pre- vs post-session) was added as a binary predictor (*Assessment*), and *Session* and *ID* were included as random effects. For the analysis of plasma and saliva oxytocin for MBGT + TAU, within-group changes were analysed using paired *t*-tests by comparing pre- and post-session levels at sessions one and four (T0–T1, T6–T7). In contrast, between-group analysis was conducted by comparing levels before session one and after session four, respectively (T0, T7).

To explore the relationships between different biological and psychological stress markers, oxytocin (plasma and saliva), cortisol, and psychological stress levels (general stress and symptom-related distress) were analysed using Spearman’s rank correlation, given the ordinal format of the self-report scales and slightly right-skewed distributions of cortisol and plasma oxytocin. The correlation analyses were additionally performed with a Holm-Bonferroni corrected significance level to correct for multiple testing.

To gain an understanding of the association between changes in stress parameters during the intervention on negative symptoms, the change from T0 to T7 in general stress (∆gStress), symptom-related distress (∆srdStress), and cortisol (∆Cortisol) was operationalised by calculating the mean change scores within each session and then averaging these values across all four sessions. The change in rater-based and self-rated negative symptoms (∆NS/PANSS and ∆NS/SNS) was obtained by calculating the difference score between T0 and T7. To analyse the effects of the different stress variables on the different negative symptom scales, the dependent variables—∆NS/PANSS and ∆NS/SNS—were regressed on each of the independent variables—∆gStress, ∆srdStress, and ∆Cortisol—, in six independent linear regression models. A significance level of *α* = 0.05 was applied to all statistical analyses.

### Ethics and inclusion statement

The research was conducted at a university hospital in a cosmopolitan metropolitan region in Germany and was led by local investigators throughout all stages of the research process, including study design, implementation, data ownership, intellectual property, and authorship. The study addressed a locally relevant clinical population, as participants were primarily outpatients receiving care in the hospital’s catchment area, and treating physicians were actively involved in recruitment and study conduct. Roles and responsibilities among collaborators were defined in advance, and capacity-building considerations were taken into account where appropriate. The study protocol was approved by the ethics committee of the participating university hospital, and all procedures were carried out in accordance with institutional and international ethical standards. The research posed no foreseeable risk of stigmatisation, incrimination, or discrimination for participants. Safety was ensured at all times by trained clinical and research personnel, and no risks to researchers were identified. To promote inclusivity and scientific equity, the literature review was conducted broadly and included relevant work from diverse cultural and international contexts.

## Supplementary information

**Supplementary information**

## Data Availability

The data supporting the findings of this study are available from the corresponding author upon request.

## Code availability

The code that was written to carry out the statistical analyses can be provided by the corresponding author upon request. All statistical analyses were performed using R Statistical Software (v4.3.0; R Core Team, 2023) and R Studio (v2023.6.0.421; Posit Team, 2023).
