# Supplementary material for: Changes of psychological and biological stress parameters in individuals with schizophrenia spectrum disorders participating in a mindfulness-based group therapy
Source: Schizophrenia (Heidelb). 2026 Apr 30;12(1):42. doi: 10.1038/s41537-026-00759-6 (PMC13133398; doi:10.1038/s41537-026-00759-6)
Supplement: Supplementary file 1 — Supplementary information [file 41537_2026_759_MOESM1_ESM.docx]

**Supplementary information**

**Supplementary Table 1**

*Descriptive Statistics of Measurement Instruments at Baseline (T0) and Post-Intervention (T7)*

|  | **MBGT+TAU (n = 22)** | | | | **TAU (n = 23)** | | | |
| --- | --- | --- | --- | --- | --- | --- | --- | --- |
|  | Mean (SD) | | Min - Max | | Mean (SD) | | Min - Max | |
| **Scale** | Baseline | Post | Baseline | Post | Baseline | Post | Baseline | Post |
| **PANSS-NS** | **20.32 (4.91)** | **19.18 (6.43)** | **9 - 28** | **8-34** | **19.3 (5.78)** | **19.61 (6.77)** | **11 - 30** | **10 - 34** |
| *Blunted Affect* | *3.64 (1.14)* | *3.27 (1.42)* | *1 - 6* | *1 - 6* | *3.22 (1.09)* | *3.39 (1.23)* | *1 - 5* | *1 - 6* |
| *Emot. Withdr.* | *3.32 (1.25)* | *3.05 (1.25)* | *1 - 6* | *1 - 6* | *3.09 (1.31)* | *3.22 (1.28)* | *1 - 5* | *1 - 5* |
| *Poor Rapport* | *2.86 (0.99)* | *2.59 (1.05)* | *1 - 5* | *1 - 5* | *2.65 (1.03)* | *2.7 (1.22)* | *1 - 5* | *1 - 6* |
| *PA Social Withdr.* | *3.18 (1.59)* | *3.09 (1.44)* | *1 - 6* | *1 - 6* | *2.96 (1.4)* | *3.0 (1.54)* | *1 - 6* | *1 - 6* |
| *Difficulty Abstract Thinking* | *2.95 (1.5)* | *2.86 (1.49)* | *1 - 5* | *1 - 6* | *2.7 (1.18)* | *2.96 (1.55)* | *1 - 5* | *1 - 6* |
| *Lack of Spontan.* | *2.09 (1.27)* | *2.23 (1.41)* | *1 - 5* | *1 - 5* | *2.52 (1.7)* | *2.26 (1.42)* | *1 - 6* | *1 - 6* |
| *Stereot. Thinking* | *2.27 (1.24)* | *2.09 (1.15)* | *1 - 4* | *1 - 5* | *2.17 (1.34)* | *2.09 (1.12)* | *1 - 5* | *1 - 4* |
| **SNS** | **15.86 (7.62)** | **14.77 (8.55)** | **3 - 30** | **0-34** | **11.78 (6.97)** | **11.96 (6.19)** | **1 - 27** | **0 - 25** |
| *Social Withdr.* | *3.32 (2.46)* | *2.68 (2.25)* | *0 - 8* | *0 - 8* | *1.7 (1.74)* | *1.78 (1.57)* | *0 - 7* | *0 - 6* |
| *Dimin. Emot. Range* | *2.5 (2.13)* | *2.86 (2.36)* | *0 - 7* | *0 - 8* | *2.26 (1.45)* | *2.26 (1.89)* | *0 - 5* | *0 - 6* |
| *Avolition* | *3.55 (2.32)* | *3.59 (2.48)* | *0 - 7* | *0 - 7* | *2.74 (2.47)* | *2.83 (2.19)* | *0 - 7* | *0 - 7* |
| *Anhedon.* | *4.18 (2.28)* | *3.41 (2.58)* | *0 - 8* | *0 - 8* | *3.22 (2.47)* | *3 (1.95)* | *0 - 8* | *0 - 8* |
| *Alogia* | *2.32 (2.15)* | *2.23 (2.14)* | *0 - 8* | *0 - 8* | *1.87 (1.6)* | *2.09 (1.44)* |  | *0 - 4* |

*Note.* MBGT = Mindfulness-based Group Therapy. TAU = Treatment As Usual. SD = Standard Deviation; Min = Minimum; Max = Maximum; Post = Post-Intervention; PANSS-NS = Negative Scale of Positive and Negative Syndrome Scale; SNS = Self-Evaluation of Negative Symptoms. Subscales are marked in italic. *Emot. Withdr.* = Emotional Withdrawal, *PA* *Social Withdr.* = Passive-apathic Soical Withdrawal, *Difficulty Abstract Thinking* = Difficulty in Abstract Thinking, *Lack of Spontan. =* Lack of Spontaneity and Flow of Conversation, *Stereot. Thinking* = Stereotyped Thinking, *Social Withdr*. = Social Withdrawal, *Dimin. Emot. Range* = Diminished Emotional Range, *Anhedon.* = Anhedonia.

**Supplementary Table 2**

*First and Second Antipsychotic Medication of Each Participant at Baseline (T0) and Post-Intervention (T7)*

|  | MBGT+TAU | | | |  | TAU | | | |
| --- | --- | --- | --- | --- | --- | --- | --- | --- | --- |
|  | APM 1 (dose) | | APM 2 (dose) | |  | APM 1 (dose) | | APM 2 (dose) | |
|  | T0 | T7 | T0 | T7 |  | T0 | T7 | T0 | T7 |
| 1 | Paliperidon (150) | Paliperidon (150) | Promethazin (50) | Promethazin (50) | 23 | Paliperidon (100) | Paliperidon (100) | / | / |
| **2** | Aripiprazol (17.5) | / | Asenapin (5) | / | 24 | Aripiprazol (*NA*) | Aripiprazol (*NA*) | Quetiapin (150) | Quetiapin |
| 3 | Aripiprazol (10) | Aripiprazol (10) | Quetiapin (500) | Quetiapin (500) | 25 | Aripiprazol (*NA*) | Aripiprazol (*NA*) | Paliperidon (*NA*) | Paliperido(*NA*) |
| 4 | Aripiprazol (20) | Aripiprazol (20) | / | / | 26 | Aripiprazol (12.5) | Aripiprazol (12.5) | / | / |
| **5** | Aripiprazol (*NA*) | **Amisulprid** (*NA*) | Amisulprid (*NA*) | **Aripiprazol** (*NA*) | 27 | Aripiprazol (7.5) | Aripiprazol (7.5) | / | / |
| 6 | Aripiprazol (20) | Aripiprazol (20) | / | / | **28** | Aripiprazol (25) | Aripiprazol **(20)** | / | / |
| 7 | Risperidon (2,5) | Risperidon (2,5) | Quetiapin (500) | Quetiapin (500) | 29 | Risperidon (7) | Risperidon (7) | Quetiapin (150) | Quetiapin (150) |
| **8** | Risperidon (1,5) | Risperidon **(1)** | Olanzapin (15) | Olanzapin **(14)** | 30 | Risperidon (0.5) | Risperidon (0.5) | / | / |
| 9 | Risperidon (3) | Risperidon (3) | / | / | 31 | Risperidon (1) | Risperidon (1) | / | / |
| 10 | Amisulprid (400) | Amisulprid (400) | Quetiapin (200) | Quetiapin (200) | 32 | Amisulprid (400) | Amisulprid (400) | Olanzapin (20) | Olanzapin (20) |
| **11** | Amisulprid (400) | Amisulprid **(600)** | Aripiprazol (10) | Aripiprazol (10) | 33 | Amisulprid (200) | Amisulprid (200) | / | / |
| **12** | Quetiapin (450) | Aripiprazol **(300)** | Aripiprazol (400) | Quetiapin **(350)** | **34** | Amisulprid (100) | Amisulprid (100) | Quetiapin (50) | Quetiapin **(25)** |
| 13 | Olanzapin (10) | Olanzapin (10) | Melperon (*NA*) | Melperon (*NA*) | 35 | Amisulprid (400) | Amisulprid (400) | / | / |
| 14 | Olanzapin (*NA*) | Olanzapin (*NA*) | / | / | **36** | Quetiapin (600) | Quetiapin **(800)** | / | / |
| 15 | Clozapin (300) | Clozapin (300) | / | / | 37 | Quetiapin (200) | Quetiapin (200) | / | / |
| 16 | Clozapin (250) | Clozapin (250) | Amisulprid (300) | Amisulprid (300) | **38** | Quetiapin (400) | / | Olanzapin (7.5) | **/** |
| 17 | Clozapin (250) | Clozapin (250) | Aripiprazol (15) | Aripiprazol (15) | 39 | Quetiapin (650) | Quetiapin (650) | Risperidon (3.5) | Risperidon (3.5) |
| 18 | Clozapin (300) | Clozapin (300) | Risperidon (37.5) | Risperidon (37.5) | 40 | Olanzapin (10) | Olanzapin (10) | Aripiprazol (20) | Aripiprazol (20) |
| **19** | Clozapin (250) | Clozapin (250) | Risperidon (2) | Risperidon **(2.125)** | 41 | Clozapin (375) | Clozapin (375) | / | / |
| 20 | Clozapin (150) | Clozapin (150) | Risperidon (3) | Risperidon (3) | 42 | Clozapin (200) | Clozapin (200) | Amisulprid (200) | Amisulprid (200) |
| 21 | / | / | / | / | 43 | Clozapin (400) | Clozapin (400) | Amisulprid (600) | Amisulprid (600) |
| 22 | / | / | / | / | 44 | Clozapin (75) | Amisulprid (100) | Amisulprid (100) | Clozapin (75) |
|  |  |  |  |  | 45 | / | / | / | / |

*Note.* MBGT = Mindfulness-Based Group Therapy. TAU = Treatment As Usual. APM = Antipsychotic Medication. T0 = Baseline. T7 = Post-Intervention. *NA* = Missing value. Medication dose is written in parantheses. Participants with changes in substance or dose of either first or second antipsychotic medication are marked in bold.

**Supplementary Table 3**

*Mean Values at Each Assessment Point of General Stress, Symptom-Related Distress, Cortisol, Oxytocin Saliva, and Oxytocin Plasma in the Active Group*

|  |  | M (SD) | | | | | | | | |
| --- | --- | --- | --- | --- | --- | --- | --- | --- | --- | --- |
| **Stress Measure** |  | **T0** | **T1** | **T2** | **T3** | **T4** | **T5** | **T6** | **T7** | |
| **General Stress** |  | 3.55  (1.26) | 1.91 (1.38) | 2.86 (1.2) | 1.86 (1.64) | 2.59 (1.62) | 1.89 (1.6) | 2.86 (1.58) | 1.59 (1.33) | |
| **Symptom-related Distress** |  | 2.5  (1.47) | 1.95 (1.46) | 2.29 (1.68) | 1.68 (1.75) | 2.29 (1.86) | 1.17 (1.61) | 2.27 (1.93) | 1.50 (1.44) | |
| **Cortisol** |  | 6.38  (4.71) | 5.02 (2.89) | 6.41 (4.23) | 4.23 (2.84) | 6.84 (4.07) | 4.34 (3.3) | 6.03 (3.43) | 4.52 (5.09) | |
| **Oxytocin Saliva** |  | 0.95 (0.09) | 1.04 (0.12) |  |  |  |  | 1.08 (0.09) | 1.00 (0.10) | |
| **Oxytocin Plasma** |  | 1.97 (0.30) | 2.05 (0.43) |  |  |  |  | 1.935 (0.19) | 1.926 (0.29) | |
| *Note.* M = mean; SD = standard deviation. T0-T7 = timepoints. | | | | | | | | | |  |

**Supplementary Table 4**

*Results of Linear Mixed Model Analysis: Effects of Assessment (Pre- vs. Post-Session) on General Stress, Symptom-Related Distress, and Cortisol*

| ***General Stress*** | | | | | | | |
| --- | --- | --- | --- | --- | --- | --- | --- |
| **Effect** | **Group** | **Term** | **Beta (Std. B)** | **SE** | **t** | **F** | ***p*-value** |
| Fixed |  | (Intercept) | 2.997 | 0.256 | 11.725 | 25.47 |  |
| Fixed |  | Assessment post | -1.201  (-0.377) | 0.159 | -7.532 | 142.73 | <.001*** |
| Random | Session:ID | SD (Intercept) | 0.000 |  |  |  |  |
| Random | ID | SD (Intercept) | 1.073 |  |  |  |  |
| Random | Residual | SD (Observ.) | 1.026 |  |  |  |  |
| ***Symptom-related Distress*** | | | | | | | |
| **Effect** | **Group** | **Term** | **Beta (Std. B)** | **SE** | **t** | **F** | ***p*-value** |
| Fixed |  | (Intercept) | 2.387 | 0.295 | 8.101 | 24.02 |  |
| Fixed |  | Assessment post | -0.675  (-0.193) | 0.154 | -4.377 | 82.52 | <.001  *** |
| Random | Session:ID | SD (Intercept) | 0.449 |  |  |  |  |
| Random | ID | SD (Intercept) | 1.26 |  |  |  |  |
| Random | Residual | SD (Observ.) | 0.991 |  |  |  |  |
| ***Cortisol*** | | | | | | | |
| **Effect** | **Group** | **Term** | **Beta (Std. B.)** | **SE** | **t** | **F** | **p-value** |
| Fixed |  | (Intercept) | 6.402 | 0.611 | 10.479 | 27.071 |  |
| Fixed |  | Assessment post | -1.990  (-0.245) | 0.418 | -4.763 | 78.313 | <.001  *** |
| Random | Session:ID | SD (Intercept) | 1.459 |  |  |  |  |
| Random | ID | SD (Intercept) | 2.384 |  |  |  |  |
| Random | Residual | SD (Observ.) | 2.588 |  |  |  |  |
| *Note.* Std. B. = Standardized effect in parentheses*.* SE = Standard Error; SD = Standard Seviation; Observ. = Observation; * *p* < .05, ** *p* < .01, *** *p* < .001. | | | | | | | |

**Supplementary Figure 1**

*CONSORT Participant Flow Chart*
